# Composition and diversity of gut microbiota across developmental stages of *Spodoptera frugiperda* and its effect on the reproduction

**DOI:** 10.3389/fmicb.2023.1237684

**Published:** 2023-09-18

**Authors:** Junrui Fu, Junhan Wang, Ximei Huang, Boyang Guan, Qili Feng, Huimin Deng

**Affiliations:** ^1^Guangdong Key Laboratory of Insect Developmental Biology and Applied Technology, Guangzhou Key Laboratory of Insect Development Regulation and Application Research, Institute of Insect Science and Technology and School of Life Sciences, South China Normal University, Guangzhou, China; ^2^Guangdong Laboratory for Lingnan Modern Agriculture, Guangzhou, China

**Keywords:** *Spodoptera frugiperda*, gut microbiota, microbiome, 16S rDNA, reproduction

## Abstract

**Introduction:**

*Spodoptera frugiperda* is a serious world-wide agricultural pest. Gut microorganisms play crucial roles in growth, development, immunity and behavior of host insects.

**Methods:**

Here, we reported the composition of gut microbiota in a laboratory-reared strain of *S. frugiperda* using 16S rDNA sequencing and the effects of gut microbiota on the reproduction.

**Results:**

Proteobacteria and Firmicutes were the predominant bacteria and the taxonomic composition varied during the life cycle. Alpha diversity indices indicated that the eggs had higher bacterial diversity than larvae, pupae and adults. Furthermore, eggs harbored a higher abundance of *Ralstonia, Sediminibacterium* and microbes of unclassified taxonomy. The dynamics changes in bacterial communities resulted in differences in the metabolic functions of the gut microbiota during development. Interestingly, the laid eggs in antibiotic treatment groups did not hatch much due to the gut dysbacteriosis, the results showed gut microbiota had a significant impact on the male reproduction.

**Discussion:**

Our findings provide new perspectives to understand the intricate associations between microbiota and host, and have value for the development of *S. frugiperda* management strategies focusing on the pest gut microbiota.

## 1. Introduction

During the evolution, insects have harbor diverse microorganisms in the gut, providing their host with physiological and ecological advantages (Philipp and Nancy, 2013; Jang and Kikuchi, 2020). Gut microbes have been recognized as a virtual “organ,” which substantially impacts on the nutrition, development, life span, reproductive capacity, defense and immune responses (Spor et al., 2011; Engel and Moran, 2013; Douglas, 2015; Schwab et al., 2016; Akami et al., 2019). For instance, *Pantoea agglomerans* in the gut of the locust *Schistocerca gregaria* produces aggregation pheromone by breaking down dietary ingredients to affect the locust aggregation (Dillon et al., 2002). *Lactobacillus plantarum* contributes to the systemic growth of *Drosophila melanogaster* (Storelli et al., 2011). Removal of the gut bacteria represses oogenesis and expedites maternal-to-zygotic-transition in the offsprings of *D. melanogaster* (Elgart et al., 2016).

It has been demonstrated that diversity of gut microbiota in insects can be modulated by many factors, including diet type (Franzini et al., 2016), host taxonomy (Kolasa et al., 2019; Huang et al., 2021), habitats environment (Ng et al., 2018), and social interactions (Martinson et al., 2012). Besides, the community structure of gut microbiota could also be altered during the different life stages of host insects (Chen et al., 2016). For instance, Proteobacteria was observed to be the most dominant phylum in eggs, pupae and adults in *Zeugodacus tau* while Firmicutes was the most dominant in the larvae (Noman et al., 2021). Moreover, feeding habits significantly impact on microbial diversity as reported in *Heliothis virescens* whereby the laboratory-reared population bacterial diversity was significantly different from that of the field-collected population (Staudacher et al., 2016). Isogenic *D. melanogaster* fed on different diets have the different microbial communities, and then distantly related drosophilids fed on the same medium have the similar microbiome (Chandler et al., 2011). Changes in the composition and structure of the gut microbiota in turn have a vital impact on host insects. Therefore, interactions between gut microbiota and host affect insect lifespan and population dynamics.

*Spodoptera frugiperda* (Lepidoptera: Noctuidae) is a world-wide agricultural pest that causes serious economic losses each year due to its hyperphagus ability on major crops. The pest has caused maize yield losses up of to 18 million tons/year and economic losses of up to 13 million US$ in 12 African countries (Harrison et al., 2019), it generally damages maize ranging from 26.50 to 70% (Lestari et al., 2020) in Indonesia, reaching 47.84% in Bali (Supartha et al., 2021), and even reaching to 100% in East Nusa Tenggara (Mukkun et al., 2021). In China, the potential economic losses caused by *S. frugiperda* to maize can reach 5.4 billion to 47 billion US$ every year (Qin et al., 2020). Moreover, *S. frugiperda* has strong migration and dispersal ability, and robust reproductive capacity (He et al., 2021; Sun et al., 2021). Hence to mitigate against losses attributed to the pest, farmers resulted to the intensive application of chemical pesticides. However, this chemotherapy continues to pose risks to the quality of yield, leads to environmental contamination and is believed to contribute to selective pressure on *S. frugiperda* in developing resistance against synthetic pesticides (Ingber et al., 2018; Gutiérrez-Moreno et al., 2019). Thus, alternative methods for *S. frugiperda* control are urgently needed, and the effects of gut microbiota on host insects have now provided new perspectives for the development of new strategies for pest control. However, detailed understanding of the roles of *S. frugiperda* associated gut microflora has to be undertaken.

In recent years, there has been an increasing number of studies on the gut microbial diversity of *S. frugiperda* from different environments or fed on different diets (Gomes et al., 2020; Rozadilla et al., 2020; Ugwu et al., 2020; Chen et al., 2021, 2022; Higuita Palacio et al., 2021; Lv et al., 2021; Ye et al., 2021). However, these studies mainly focused on the gut microbial community associated with a certain stage of larvae in *S. frugiperda*, providing only a single snapshot of the bacterial community. Furthermore, due to gut remodeling during metamorphosis, we hypothesize that gut microbial communities may differ across the developmental stages. Recently, several studies revealed the dynamics of microbial communities in *S. frugiperda* during its life cycle (Li et al., 2022; Chen et al., 2023; Lü et al., 2023), showing that the environment and diet can affect the gut microbial community in *S. frugiperda*.

This study focused on the abundance and diversity of gut microbiota across developmental stages (eggs, larvae, male and female pupae, male and female adults) of laboratory-reared *S. frugiperda* using high-throughput sequencing of 16S rDNA amplicons. Furthermore, the effect of gut bacteria on the development and reproduction was studied by using continuous antibiotic treatment. Hence, the findings of our study contribute to the understanding of some key roles of the gut micro flora in *S. frugiperda*, giving insights into the management of the pest.

## 2. Materials and methods

### 2.1. Insect rearing

A laboratory population of *S. frugiperda* originally collected from corn fields in Dali city, Yunnan Province, China, was established and maintained in our laboratory. After hatching from eggs, *S. frugiperda* larvae were reared on artificial diet without antibiotics (soybean powder 100 g, wheat bran 80 g, yeast powder 26 g, agar 26 g, casein 8 g, cholesterol, inositol 0.2 g, sorbic acid 2 g, choline chloride 1 g, vitamin C 8 g and distilled water 1000 mL) as described previously (Jia et al., 2009). All larvae and adults were reared under the conditions of 26 ± 1°C, 65% ± 5% relative humidity, and a photoperiod of 14:10 h (Light:Dark). The emerged adults were supplied with 10% honey solution.

### 2.2. Sample collection and 16S rDNA sequencing

Larvae (4th and 6th instar), 2-day-old pupae (female and male), 2-day-old virgin adults (female and male) and fresh egg masses of next generation (<12 h) were kept on ice prior to dissection. All insects were firstly rinsed in sterile 1 × PBS solution (pH7.4), surface sterilized using 75% ethanol for 30 s and rinsed again in sterile PBS. The whole gut tissue was dissected from each individual. Egg masses were not surface-sterilized. All gut samples were stored at −80°C prior to DNA extraction and later 16S rDNA sequencing for bacterial analysis. A total of 15 larvae (4th and 6th instar), 15 pupae (female and male), 15 adults (female and male) and 300 eggs were processed for gut bacterial 16S rDNA sequencing. Each treatment group consisted of 3 biological replicates.

Bacterial DNA was extracted using the HiPure Soil DNA Kits (Magen, Guangzhou, China) according to the manufacturer’s protocols. The hypervariable V3-V4 region of the 16S rDNA gene was amplified using the primers 341F (5′-CCTACGGGNGGCWGCAG-3′) and 806R (5′-GGACTACHVGGGTATCTAAT-3′) (Guo et al., 2017). PCR enrichment was performed in a 50 μL reaction containing a 50 ng template, a fusion PCR primer (341F and 806R), and a PCR master mix (Vazyme). PCR cycling conditions were as follows: 95°C for 5 min, 30 cycles of 95°C for 30 s, 56°C for 45 s, 72°C for 1 min, and a final extension at 72°C for 10 min. Amplicons were excised from 2% agarose gels and purified using the AxyPrep DNA Gel Extraction Kit (Axygen Biosciences, Union City, CA, USA) according to the manufacturer’s instructions and quantified using ABI StepOnePlus Real-Time PCR System (Life Technologies, Foster City, USA). DNA library sequencing (250 bp read length) was performed on the Illumina Novaseq 6000 platform by Gene *Denovo* Biotechnology Co., Ltd., (Guangzhou, China). The raw reads were deposited into the NCBI Sequence Read Archive (SRA) database.

### 2.3. Effects of gut microbiota on growth and reproduction

To investigate the influence of gut bacteria on the growth and reproductive capacity, antibiotics were used to treat larvae. Egg masses were collected 48 h after laying and dechorionated for 4 min in 4% formaldehyde solution, then immersed in 8% sodium hypochlorite solution (containing 4% sodium hydroxide) for 3 min, and rinsed twice with sterile water (Elgart et al., 2016). The eggs were then transferred to the sterile solid medium of beef extract peptone (beef extract 3 g, peptone 10 g, NaCl 5 g, ddH_2_O 1000 mL, pH 7.2∼7.4) and allowed to develop. When the media was confirmed to be free of bacterial contamination, the newly hatched larvae were transferred to sterile artificial diet that was mixed with a combination of antibiotics (gentamycin, penicillin, streptomycin, ciprofloxacin hydrochloride, rifampicin and vancomycin, each at 100 μg/mL diet). To maintain sterile conditions, consumables and equipment were steam sterilized through autoclaving and all manipulations were performed in a biosafety cabinet. The adults were fed on fresh 10% sterile honey solution mixed with the above cocktail of antibiotics. Next generation eggs were adhered to sterile spawning papers, and called first generation (G1) post antibiotic treatments. The G1 insects were raised under the same conditions. The effects of gut microbiota on the growth and reproduction of *S. frugiperda* were analyzed by measuring the body weight, developmental period of G1, and the hatching rate of eggs laid by G1 adults. A total of 30 insects per treatment group were used to measure the weight and developmental period, and 300 eggs were assessed for hatching rate in each treatment. All insects were reared in the same rearing bottles and environmental conditions. In addition, the effects of gut microbiota on reproduction were further verified different mating combinations between control adults and treated adults. At the same time, fresh feces of sixth-instar larvae were collected, 500 uL of sterile water was added, then the mixture was homogenized at 150 rpm for 2 h at 28°C on a rotary shaker. Thereafter, the mixture was allowed to settle for 10 min, then 100 μL of the supernatant was taken out and added into 100 mL of non-solidified sterile diet without antibiotics and mixed evenly. The G1 generation was fed with the diet mixed with fresh fecal bacteria from larvae to adults.

### 2.4. Bioinformatics and data analysis

After 16S rDNA sequencing, raw reads were filtered to remove adaptors and lower quality bases using FASTP (version 0.18.0; Chen et al., 2018), then paired end clean reads were merged as raw tags using FLASH (version 1.2.11) with a minimum overlap of 10 bp and mismatch error rates of 2%. The clean tags were clustered into operational taxonomic units (OTUs) of ≥ 97% similarity using the UPARSE pipeline (version 9.2.64) (Edgar, 2013). All chimeric tags were removed using the UCHIME algorithm (Edgar et al., 2011) and non-chimeric tags were then used for further analyses. The tag sequence with the highest abundance was selected as a representative sequence within each cluster. The representative OTU sequences were classified into organisms by a naïve Bayesian model using RDP classifier (version 2.2; Wang et al., 2007) based on the SILVA database (version 132; Pruesse et al., 2007), with the confidence threshold value of 0.8.

### 2.5. Bacterial composition and diversity analysis

Community diversity of the gut was estimated using alpha diversity including the Chao1 and Shannon indices using QIIME (version 1.9.1; Caporaso et al., 2010). Differences in the bacterial community among the tested stages (larva, pupa, adult and egg) were calculated with principal coordinates analysis (PCoA) and analysis of similarity (ANOSIM) based on the Weighted_Unifrac distances using the filtered and rarefied OTUs. Linear discriminant analysis coupled with effect size (LEfSe) analysis (LDA score > 2, *P* < 0.05) was used to identify the OTUs characteristic of each of the groups (Segata et al., 2011). Bar plots and heatmaps were generated to visualize the taxonomic diversity (at different levels) found in the total OTUs of the various developmental, using the R package gplots (version 3.0.1; Warnes et al., 2016). To identify statistically significant differences among the groups, Wilcoxon rank-sum test and Kruskal–Wallis H test were performed by Graphpad (v. 9.4.1; citation) and *P* < 0.05 was considered as statistical significance.

### 2.6. Function prediction

To predict the metabolic functional profiles of the bacterial communities in each of the developmental stages, PICRUSt2 software was used to predict microbial functions and functional pathways based on the evolutionary genealogy of genes from different databases (Toole et al., 2021). Microbiome phenotypes were classified using the BugBase (Ward et al., 2017). Function difference between the groups was calculated by Welch’s *t*-test, and Kruskal–Wallis H test in the R project Vegan package (version 2.5.3; Oksanen et al., 2011).

## 3. Results

### 3.1. 16S rDNA sequencing data

**FIGURE 1:**
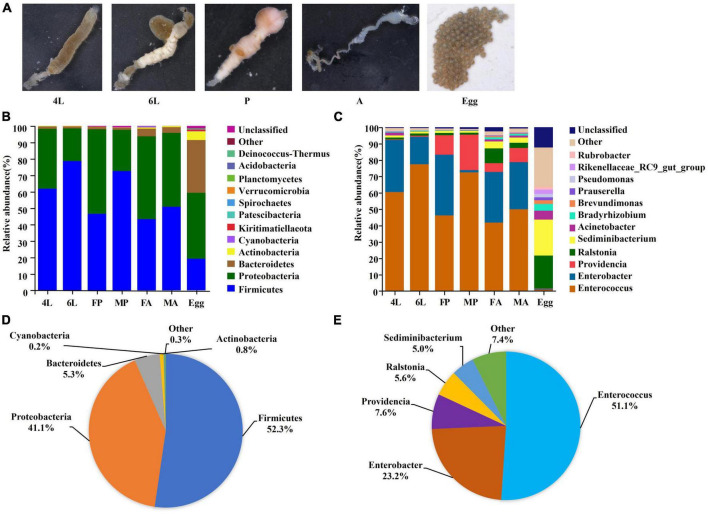
Composition of gut microorganism across the life cycle of *S. frugiperda*. **(A)** Gut samples collected from different developmental stages. **(B)** Top 14 of in relative abundance of microorganism composition at the phylum. **(C)** Top 14 of in relative abundance of microorganism composition at genus level. **(D)** Composition at phylum level for all samples merged and for developmental stages. **(E)** Composition at class level for all samples merged and for developmental stages. 4L, 4th instar larvae; 6L, 6th instar larvae; FP, female pupae; MP, male pupae; FA, female adults; MA, male adults.

The guts from different stages reared on artificial diet, and fresh egg masses of next generation (<12 h) (Figure 1A) were used for 16S rDNA sequencing, respectively. A total of 2,761,310 raw reads were obtained and 2,758,386 clean reads were generated. The total OTUs was 3,292 from all samples. The observed gut microorganisms were classified into 25 phyla, 43 classes, 81 orders, 130 families and 259 genera (Supplementary Table 1). The total amount of sequences generated from each stage, as well as the length of the analyzed sequences of each stage were also analyzed (Supplementary Table 2). Sufficient sequencing data were obtained based on the plateaued rarefaction curves, and Good’s coverage of all samples was above 99%, indicating that the sequencing data was sufficient to fully estimate the bacterial diversity of *S. frugiperda* (Supplementary Figure 1).

There were significant differences in unique OTUs in male adults and male pupae (*P* = 0.0132) whereby we observed male adults to have more unique OTUs as compared to the male larvae. Moreover, a similar difference (*P* = 0.0054) was observed in female adults and fourth instar larvae (Supplementary Figure 2).

### 3.2. Changes in the gut microbial composition at different developmental stages

To investigate the variability of *S. frugiperda* bacterial communities at different developmental stages, the top 14 most abundant phyla and genera were analyzed. The dominant bacterial phyla associated with *S. frugiperda* over the whole life cycle were Firmicutes (52.3%) and Proteobacteria (41.1%), followed by Bacteroidetes (5.3%) and Actinobacteria (0.8%) in the whole life cycle (Figures 1B, D). Proteobacteria accounted for over 50% of all bacterial phyla in female pupae and adults, while Firmicutes was more abundant in 6th instar larvae (78.6%) and male pupae (72.6%) (Figure 1B). At the genus level, *Enterococcus* (51.1%) was the most abundant, followed by *Enterobacter* (23.2%), *Providencia* (5.37.6%), *Ralstonia* (5.6%) and *Sediminibacterium* (5%) (Figures 1C, E). Within Firmicutes, the genus *Enterococcus* dominated in the larvae, pupae and adults, and the abundance of this genus was significantly greater in the 6th instar larvae (77.5% average abundance) (Figure 1C). However, the genera *Ralstonia* (20%) and *Sediminibacterium* (22%) were more abundant in the next generation eggs (Figure 1C). These results indicate that the bacterial community members change with the developmental stages.

### 3.3. Comparison of the microbial diversity in the guts at different developmental stages

**FIGURE 2:**
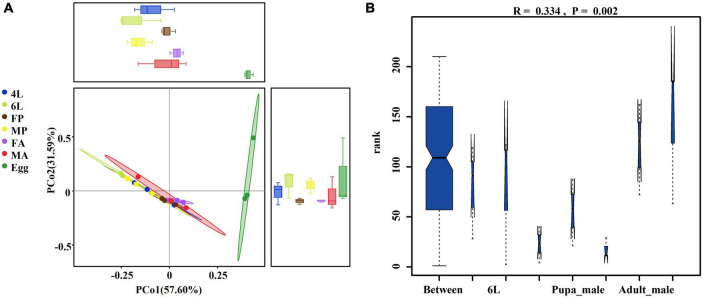
Principal component and non-parametric test analysis of bacterial diversity. **(A)** Two-dimensional PCoA of microbial communities based on β-diversity metrics by using the Weighted-unifrac calculation to measure different stage samples. **(B)** Non-parametric test (ANOSIM) of bacterial diversity among different life stage (*r* = 0.0.334, *P* = 0.002). 4L, 4th instar larvae; 6L, 6th instar larvae; FP, female pupae; MP, male pupae; FA, female adult; MA, male adults.

To further detect changes in gut microbiota in different developmental stages, the principal co-ordinates analysis (PCoA) based on weighted-unifrac distance and with the OTU-level profiles were performed. The results showed a clear divergence between the egg group and the other groups. The first principal component explained 57.60% of the variation, the gut bacterial composition of female pupae and female adults was relatively concentrated, but still showed differences along the second principal components explained 31.59% of the variance. Clustering according to the gut developmental stage revealed that microbiome community structure in 4th and 6th larval instars were more closely related to each other than to other life stages (Figure 2A). To further determine whether the grouping was meaningful, non-parametric test based on the weighted-unifrac distance through ANOSIM indicated that bacterial diversity varied significantly and the grouping was reasonable (*R* = 0.334, *P* = 0.002) among the different developmental stages (Figure 2B). These results suggest that the samples from the same developmental stage are more closely related, while egg samples formed a separate cluster.

**FIGURE 3:**
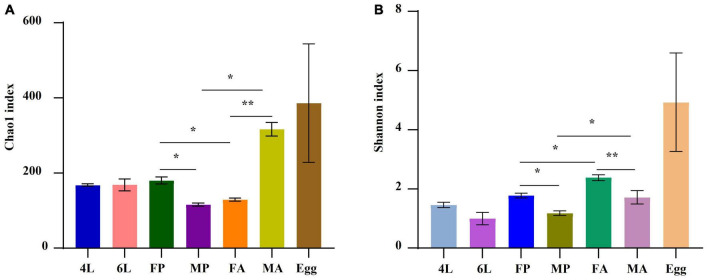
Distribution of alpha diversity at different developmental stages measured by panels **(A,B)** Chao1 and Shannon index. The metrics were based on the total OTUs. Each color represents a developmental stage. Error bars indicate ± SE (*P* < 0.05, *t*-test). *P < 0.05. **P < 0.01. 4L, 4th instar larvae; 6L, 6th instar larvae; FP, female pupae; MP, male pupae; FA, female adult; MA, male adults.

Compared with other stages, eggs were richer in terms of species diversity (Figure 3A) and this richness was more homogeneous in the eggs (Figure 3B). Clear differences in both gut bacterial richness and evenness were observed between the pupae and adults (*P* < 0.05), and between the female adults and male adults (*P* < 0.01) (Figure 3).

### 3.4. The core microbiotic species across different developmental stages

**FIGURE 4:**
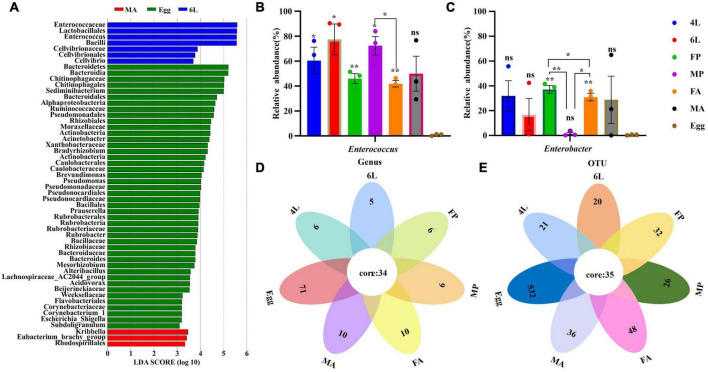
Significant difference analysis of microorganism community across the life cycle of *S. frugiperda*. **(A)** Notable differences of gut bacteria across the life stages were analyzed by LEfSe analysis at different taxonomic levels (LDA > 3). **(B,C)** Comparison of the relative abundance of *Enterococcus* and *Enterobacter* across the different life cycle, respectively. Error bars indicate ± SE (*P* < 0.05, ANOVA with Tukey’s HSD test). *P < 0.05. **P < 0.01. **(D,E)** Venn diagrams showing the overlap of the bacterial community between the different developmental stages at the genus and OUT levels, respectively. 4L, 4th instar larvae; 6L, 6th instar larvae; FP, female pupae; MP, male pupae; FA, female adult; MA, male adults.

To identify the most abundant species at different developmental stages, LDA effect size analysis (LEfSe) was performed to detected the notable differences of gut bacteria across the life stages of *S. frugiperda*. Based on non-parametric ranksum test (LDA > 3, *P* < 0.05), the results revealed that the number of taxa was, respectively 7, 3, and 44 among the 6th instar larvae, male adults and eggs, while other stages had no significant microorganisms (Figure 4A). There were more biomarker species in the eggs than that in the 4th instar larvae, female pupae, male pupae and female adults. At the genus level, *Eubacterium* and *Kribbella* were notably enriched in the male adults. *Sediminibacterium*, *Acinetobacter*, *Brevundimonas*, *Pseudomonas*, *Prauserella*, *Rubrobacter*, *Bacteroides*, *Mesorhizobium*, *Alteribacillus*, *Acidovorax*, *Escherichia*_*Shigella* and *Subdoligranulum* were enriched in the eggs, whereas *Enterococcus* was the most abundant bacteria in the 6th instar larvae (Figure 4A). The abundance of the top two genera *Enterococcus* and *Enterobacter* were varied inhabiting in the eggs, larvae, pupae, and adults (Figure 4B). Compared with the eggs, the abundance of *Enterococcus* in the larvae, pupae and female adults diversified significantly (*P* < 0.05), and the abundance of *Enterobacter* in the female and male adults were significantly different from eggs (*P* < 0.01) (Figure 4B). Similar to LEfSe analysis, *Enterococcus* was abundant in the 6th instar larvae, while *Enterobacter* was abundant in the female pupae and adults. Furthermore, sex-dependent bacterial communities were evident in both pupae and adults (Figure 4B).

The interaction in bacterial communities across *S. frugiperda* life stages is shown by Venn diagrams (Figure 4C). A total of 34 genera and 35 OTUs were present in the eggs, larvae, pupae and adults, out of which 15 genera and 18 OTUs were abundant (Supplementary Table 3). The top 12 common genera and the corresponding OTUs were *Enterococcus* (OTU000001), *Enterobacter* (OTU000003), *Providencia* (OTU000007), *Ralstonia* (OTU000002), *Sediminibacterium* (OTU000004), *Acinetobacter* (OTU000005), *Bradyrhizobium* (OTU000008), *Rubrobacter* (OTU000011), *Prauserella* (OTU000012), *Brevundimonas* (OTU000009), *Alteribacillus* (OTU000016) and *Pseudomonas* (OTU000010). We hypothesize that the above results suggest that these common gut bacterial genera may be vital for physiological activities across the whole life cycle of *S. frugiperda*.

### 3.5. Function prediction of the gut microbiome in *S. frugiperda*

**FIGURE 5:**
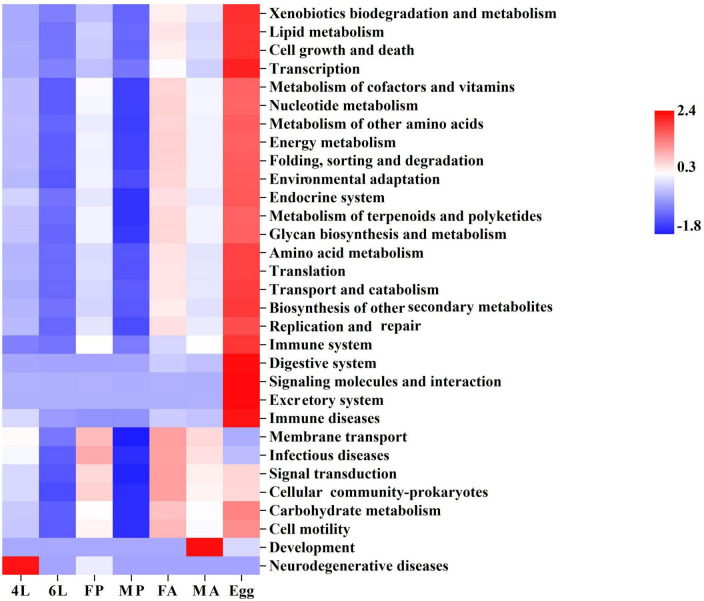
Inferred functions of bacterial community associated of *S. frugiperda*. The heatmap of Kyoto Encyclopedia of Genes and Genomes (KEGG) level-2 functions of bacteria during various developmental stages. 4L, 4th instar larvae; 6L, 6th instar larvae; FP, female pupae; MP, male pupae; FA, female adult; MA, male adults.

The potential metabolic functions of the gut bacterial communities were predicted using PICRUst2. Functional categories at the second level were most abundant in the eggs, including lipid metabolism, amino acid metabolism, cell growth and death, transcription, transport and catabolism (Figure 5). Interestingly, abundances of several categories (such as lipid metabolism, energy metabolism, environmental adaptation, amino acid metabolism and translation) showed an increasing trend from the female pupae and female adults to next generation eggs, suggesting a vertical transmission of specific bacteria from the female adults to its offspring via maternal secretions. Additionally, most microbiota functions were abundant in the male pupae, especially carbohydrate metabolism, membrane transport and signal transduction (Figure 5). Moreover, further functional classifications (top 40 abundances) at the third level indicated that phosphotransferase system (PTS) was less abundant in the eggs (Supplementary Figure 3). PTS is a major mechanism used by bacteria for uptake of carbohydrates and their derivatives through the phosphorylation cascade into the cell. Larvae and adults require gut microbiota to help digest plant fibers and absorb energy, resulting in a more enriched PTS pathway.

**FIGURE 6:**
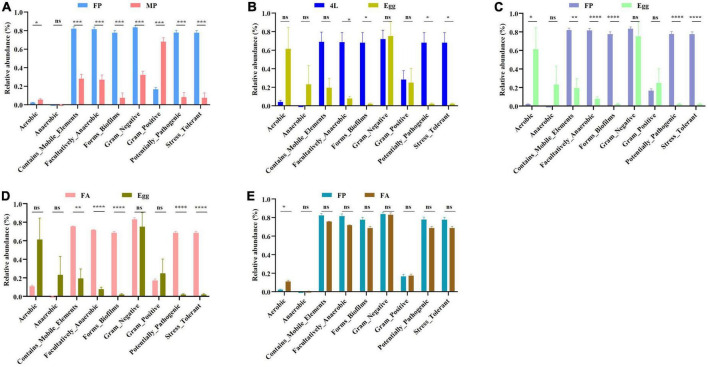
Comparative analysis of phenotype differences of bacterial community associated in *S. frugiperda*. **(A)** Phenotype difference between female pupa and male pupa. **(B)** Phenotype difference between the 4th larvae and eggs. **(C)** Phenotype difference between female pupa and eggs. **(D)** Phenotype difference between female adult and eggs. **(E)** Phenotype difference between female pupa and female adult. 4L, 4th instar larvae; 6L, 6th instar larvae; FP, female pupae; MP, male pupae; FA, female adult; MA, male adults. *P < 0.05. **P < 0.01. ***P < 0.001. ****P < 0.0001.

In addition, the bacterial phenotype for each group was analyzed using BugBase. Nine potential phenotypes, including aerobic, anaerobic, mobile elements, facultatively anaerobic, biofilm forming, gram-negative, gram-positive, potentially pathogenic, and stress tolerant were predicted (Figures 6A–E). Comparing the abundance differences of phenotypes at different developmental stages, among all the phenotypes, the relative abundances of mobile elements, facultative anaerobic, biofilm forming, gram-negative, gram-positive, potential pathogenic and stress tolerant showed significant positive relationships with female pupae and female adults, and the anaerobic phenotype presented a significant positive relationship with eggs.

### 3.6. Effect of gut bacteria on the growth, development and reproduction

**FIGURE 7:**
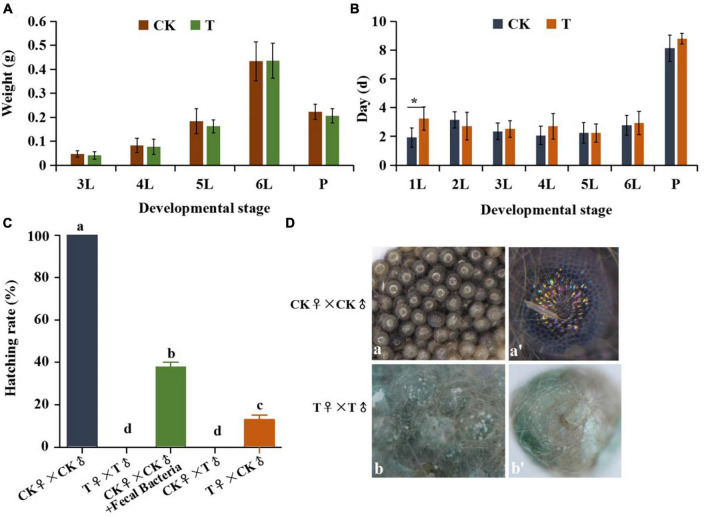
Effects of antibiotic treatment on growth, development and hatching rate of *S. frugiperda*. **(A)** Weight gain at each instar larvae and pupae. **(B)** Developmental duration of control and G1 antibiotic treated groups. **(C)** The hatching rate of eggs laid by control adults and G1 antibiotic treated adults. **(D)** Changes in the developmental morphology of eggs laid by control adults and G1 antibiotic treated adults. Error bars indicate ± SE (*P* < 0.05, ANOVA 7 with Tukey’s HSD test). Different letters above the columns indicate the significant difference. 4L, 4th instar larvae; 6L, 6th instar larvae; FP, female pupae; MP, male pupae; FA, female adult; MA, male adults; CK, control adults; T, antibiotic-treated adults. *p < 0.05.

To further determine the function of gut bacteria in *S. frugiperda*, antibiotics were added to the artificial diet for two generations, then the body weight, developmental duration of G1 and hatching rate of G2 eggs were analyzed. The results showed that there was no significant difference in body weight of larvae and pupae between the control and antibiotic treatment (*P* < 0.05) (Figures 7A, B). Comparing developmental periods at different ages, only the developmental duration (3.23 ± 0.81 d) of the first instar larvae after the antibiotic treatment was significantly longer than that of control group (1.93 ± 0.67 d) (*P* < 0.05), and there were no significant differences in subsequent developmental instars (Figure 7B). The possible explanation was that the early-hatched larvae were affected after the formaldehyde and hypochlorite treatment of eggs. At the advanced stage, the body weight and developmental process of the treated groups appeared to be lower than those of the control groups, although the effect was not significant (Figures 7A, B). These results imply that the antibiotics treatment for one generation has no significant impact on the growth and development of *S. frugiperda*.

Furthermore, while eggs laid by the control group adults had 100% hatch rate, eggs failed to hatch after two consecutive generations in sterile conditions with antibiotic treatment (Figure 7C), and abnormal embryogenesis were observed inside the eggs (Figure 7D). However, when the fecal bacteria were added to the diets, the hatching rate of eggs laid by antibiotic treated adults significantly increased (37.78%). Moreover, the eggs laid by the control group females mated with antibiotic treated males also failed to hatch, whereas the hatching rate of eggs laid by antibiotic treated female mated with control group males was 13.03% (Figure 7C). We postulate from these findings that the antibiotics treatment for two generation results in the decreased reproductive capacity of *S. frugiperda*, and has a greater effect on the male reproductive.

## 4. Discussion

Our findings have demonstrated the gut microbial abundance and diversity across all the developmental stages in laboratory reared *S. frugiperda*. Our results were in congruence with other studies on *S. frugiperda* and other Lepidopterans whereby Proteobacteria and Firmicutes were the most dominant bacterial phyla (Chen et al., 2016; Xia et al., 2018; Gichuhi et al., 2020; Li et al., 2022). In this study, we found that Firmicutes and Proteobacteria were the dominant phylum at larval, pupal and adult stages, whereas Proteobacteria and Bacteroidetes were more abundant in the eggs. Firmicutes and Proteobacteria have been reported to play key roles in the nutritional supplementation, energy absorption, preservation of gut homeostasis and host immunity (Colston and Jackson, 2016; Wang et al., 2020). Since Lepidopterans are highly phytophagous insects, their larval stages will ordinarily ingest large amounts of plant materials and other potentially harmful microbes associated with their food. On the other hand, the adults will require energy for essential activities such as migration and reproduction. Therefore, Firmicutes and Proteobacteria may play important auxiliary roles in the growth and development of *S. frugiperda* larvae and adults, as well as in fighting pathogens. In this study, the main genera of *Enterococcus* within Firmicutes and *Enterobacter* belonged to Proteobacteria were detected in *S. frugiperda* fed on artificial diet. Previous studies have showed that *Enterococcus* is able to degrade alkaloids and latex, and has a putative role in detoxifying plant toxins (Brinkmann et al., 2008; Yun et al., 2014; Gao et al., 2019; Gomes et al., 2020; Liu et al., 2020). Additionally, *Enterobacter* contributes to the synthesis of vitamins and pheromones, the degradation of plant compounds and the process of nitrogen fixation (Lilburn et al., 2001; Morales-Jiménez et al., 2012). The higher abundances of *Enterococcus* and *Enterobacter* at the larval, pupal and adult stages (Figure 1E), implies that they may contribute to *S. frugiperda* nutrient absorption. The shift in abundance of dominant genera across developmental stages reveal that they are required for each stage.

Beta and Alpha diversity analyses revealed that the eggs had the highest species abundance (Figure 2), were most homogeneous in terms of distribution and their microbial communities clustered separately from the other developmental stages (Figure 3). Moreover, there was a significant decline in bacterial diversity as the *S. frugiperda* developed from the egg to pupae. This reduction could be attributed to the reduced association between neonate larvae and the maternal bacteria (there is usually a close association between the egg mass, maternal and environmental microbiota). This phenomenon is also found in various insect species, such as *S. littoralis* (Chen et al., 2016) and *S. frugiperda* (Li et al., 2022). At the genus level, *Ralstonia* and *Sediminibacterium* were the most abundant in the eggs (Figure 1C), but their functions have not been reported in insects. The genus *Ralstonia* was also found to be the most abundant bacterium in the egg stage of *S. frugiperda* reared with maize seedlings under laboratory conditions (Li et al., 2022). However, *Ralstonia* showed a higher abundance in the 2^nd^ instar of *S. frugiperda* fed on maize plants in the laboratory (Lü et al., 2023). The genus *Sediminibacterium* belongs to the phylum Bacteroidetes and mainly exists in the diverse natural environment, such as sediment, groundwater and soil (Song et al., 2017), but it has been not reported in Lepidoptera. Moreover, the genus *Weissella* was found to be the main dominant in the 5th instar larvae, which were all purchased from Keyun Biocontrol Engineering Co., Ltd., (Jiyuan, China) and fed on artificial diet in the laboratory for several generations (Chen et al., 2021; Ye et al., 2021), but this was not detected in our study. The composition differences may be due to the bacteria in the air/on surfaces in the laboratory. Interestingly, core taxa associated with female adults were also detected in the eggs, implying that some gut symbionts are transmitted vertically between parents and offspring. This maternal transmission of core gut microorganisms to the next generation could be essential in stabilization and coevolution of host-microbe (Chen et al., 2016). Gut microbes, such as the genus *Asaia* that are vertically transmitted from parents to offsprings provide vitamins, nitrogen and amino acids for egg development (Damiani et al., 2008). Although the abundances of *Enterococcus* and *Enterobacter* were much lower in the eggs, these taxa were successfully colonized and steadily inherited in the larval gut of next generation in *S. littoralis* (Chen et al., 2016).

Diet significantly impacts the composition and functions of the gut microbiota in insects (Rinke et al., 2013; Mason et al., 2021). Artificial diets are a poorer microbial source for insects than natural plant diets (Gayatri Priya et al., 2012). *S. frugiperda* fed on artificial diet hence harbors significantly lower microbial diversity than the larvae fed with plant leaf (Lv et al., 2021; Lü et al., 2023). In *Ostrinia nubilalis* (Belda et al., 2011) and *Helicoverpa armigera* (Xiang et al., 2006), Firmicutes become the dominant gut microorganism after the larvae were fed with artificial diets. These results indicate that Firmicutes may play an important role in the gut of insect reared on the artificial diets, probably by facilitating carbohydrate and nucleotide metabolism to enhance the digestion and absorption of nutrients. The result also reflects that the compositions of the diet that the host insects feed on may dominate the structural and potential functional of the gut microbiota. Although, there are some differences in microbial composition between lab-reared and wild-caught populations of this pest. The isolation and identification of microflora in laboratory are more helpful to further validate the pest management decisions made in the field. Moreover, PTS is a major mechanism used by bacteria for uptake of carbohydrates and their derivatives through the phosphorylation cascade into the cell. Thus, the enriched PTS pathway showed that larvae and adults require gut microbiota to help digest plant fibers and absorb energy. Therefore, the results of this study are consistent with material requirements during insect growth and development.

Another interesting discovery in this study is that there was clear differences in sex-dependent microbes between males and females based on the Chao1 and Shannon index (Figure 3) and abundance differences (Figure 4B). Our results support earlier findings of sex-dependent microbiomes in *S. frugiperda* (Lü et al., 2023) and this phenomenon was also reported in black flies (Tang et al., 2012). Moreover, functional metabolic differences between males and females also showed that the metabolic functions were enhanced in females (Figure 5), and the phenotype differences were also displayed in a sex-manner (Figure 6). For *S. frugiperda*, female adults require more nutrients and energy for migration, ovarian development and reproduction (Chapman et al., 2015). Therefore, females may need more abundance of bacteria to digest food so that they can provide more nutrient and energy to the next generation.

In addition, *S. frugiperda* is a holometabolous insect, and has a metabolically dynamic and complex process during larval-to-pupal and pupal-to-adult metamorphosis, in which the gut microbiome also undergoes significant structural changes. This might be related to the reorganization of the intestinal microbiota structure from pupal to adult transition during metamorphosis of *S. frugiperda*. The gut during the transition from larvae to adult is believed to undergo a sterilization process and adults recruit new microbiota (Kohl et al., 2013). However, we found that most microbioal genera were able to survive metamorphosis and be transmitted to the emerging adults (Figure 4C). It is likely that gut microbiota plays a role in the gut remodeling process of *S. frugiperda.*

Currently, antibiotic feeding is considered the best approach for studying bacterial function in insects (Noman et al., 2021). In this study, we treated *S. frugiperda* eggs with disinfection reagents (formaldehyde and sodium hypochlorite) and fed larvae with the diet containing antibiotics mixture for two generations. The hatchability of eggs was not affected by antibacterial reagents. However, the side effect of antibiotics treatment cannot be excluded. We found that the gut microbiota dysbiosis had no significant effects on the body weight gain and developmental duration in the tested larvae, but significantly inhibited the hatching of offspring eggs. The incorporation of fecal bacteria to the diet allowed some eggs to hatch (Figure 7), implying that inhibition of some gut bacteria decrease the fecundity.

Suppressed reproduction rates have has also been found in some insects fed with antibiotics, such as *Riptortus pedestris* (Lee et al., 2017), *Zeugodacus tau* (Noman et al., 2021) and *Spodoptera litura* (Thakur et al., 2016). Unexpectedly, inhibition of gut bacteria had greater effect on the male reproduction than the female reproduction (Figure 7C), alluding that maybe male adults are more susceptible to gut microflora changes.

To date, symbiotic bacteria have been used to control economically important in management strategies. Several bacterial species, such as *Klebsiella oxytoca*, *Enterobacter cloacae*, *Pantoea agglomerans* and *Bacillus cereus* have been used for attractants and in the control of both males and females of *Bactrocera tau* and *B. dorsalis* (Prabhakar et al., 2009; Shi et al., 2012). Bacteria have a great contribution to SIT by increasing the life expectancy and mating performance of male adults of *Ceratitis capitata* (Niyazi et al., 2004). It may be a suitable technique to control *S. frugiperda* species through the combination of attractancy, SIT and application of parasitoids (Noman et al., 2019). In this study, eliminating the bacteria from the gut of *S. frugiperda* and inhibition of egg-hatching can be considered as potent options for the management of *S. frugiperda*.

In summary, we analyzed the composition and diversity of gut microbiota across the developmental stages of *S. frugiperda* fed on artificial diet without or with antibiotics, and found that *Enterococcus* was the most dominant genus for larval, pupal and adult stages, whereas *Ralstonia* and *Sediminibacterium* were dominant in the eggs. Moreover, antibiotics treatment resulted in a reduction of reproductive capacity, especially of male reproductive capacity, in *S. frugiperda.* These results provide a base and some hints to further investigate the roles of the gut microbiota in the growth, development and reproduction of insects.

## Data availability statement

The datasets presented in this study can be found in online repositories. The names of the repository/repositories and accession number(s) can be found in the article/Supplementary material.

## Author contributions

HD and QF designed and monitored the project and finalized the manuscript. JF, JW, XH, and BG performed the experiments. JF interpreted the data and wrote the manuscript. JW, XH, and BG prepared some of the experimental materials. All authors contributed to the article and approved the submitted version.
