# Supplementary figures and images for: Composition and diversity of gut microbiota across developmental stages of Spodoptera frugiperda and its effect on the reproduction
Source: Front Microbiol. 2023 Sep 18;14:1237684. doi: 10.3389/fmicb.2023.1237684 (PMC10543693; doi:10.3389/fmicb.2023.1237684)

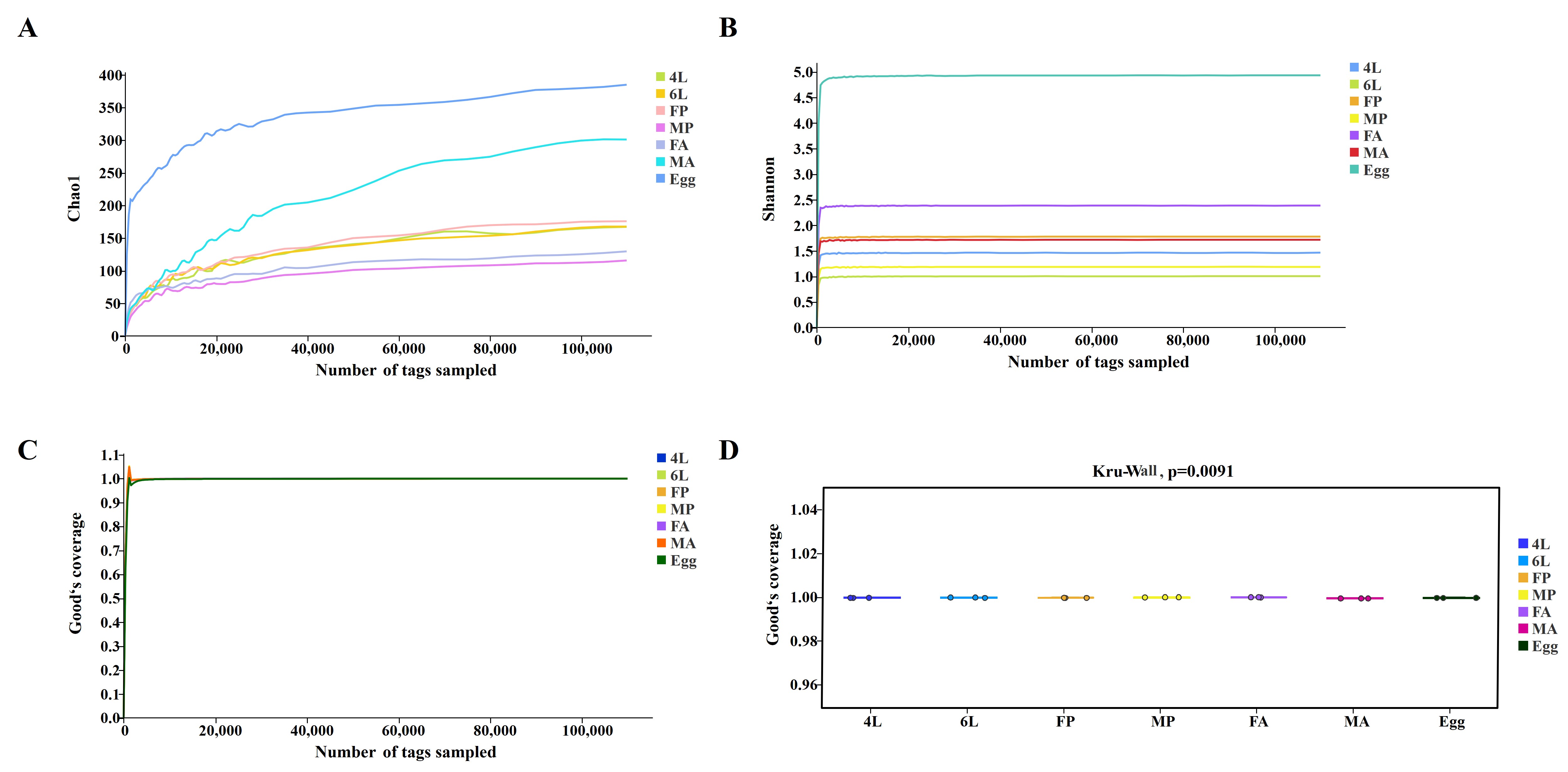

Supplement: Supplementary file 1 [file Image_1.JPEG]

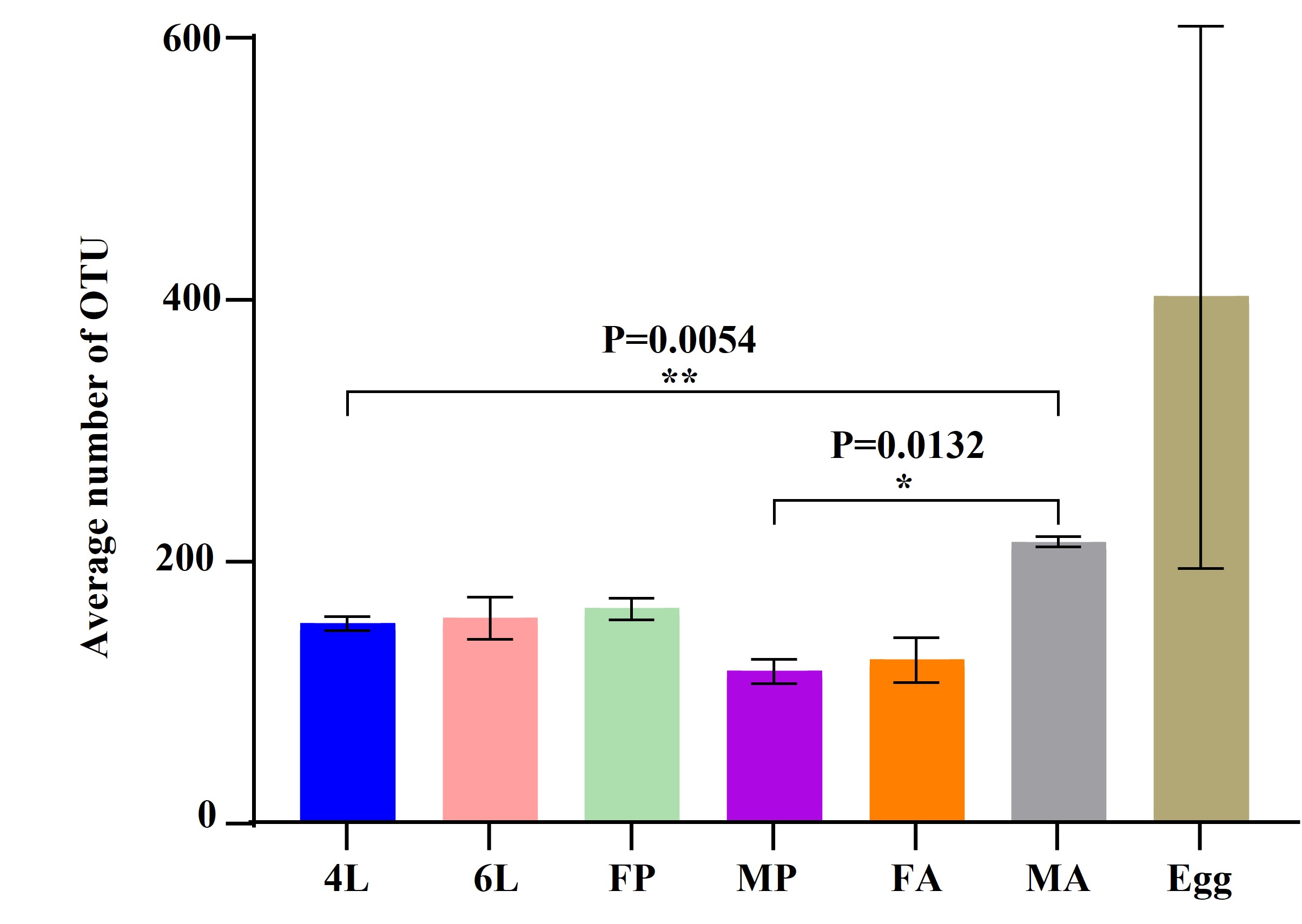

Supplement: Supplementary file 2 [file Image_2.JPEG]

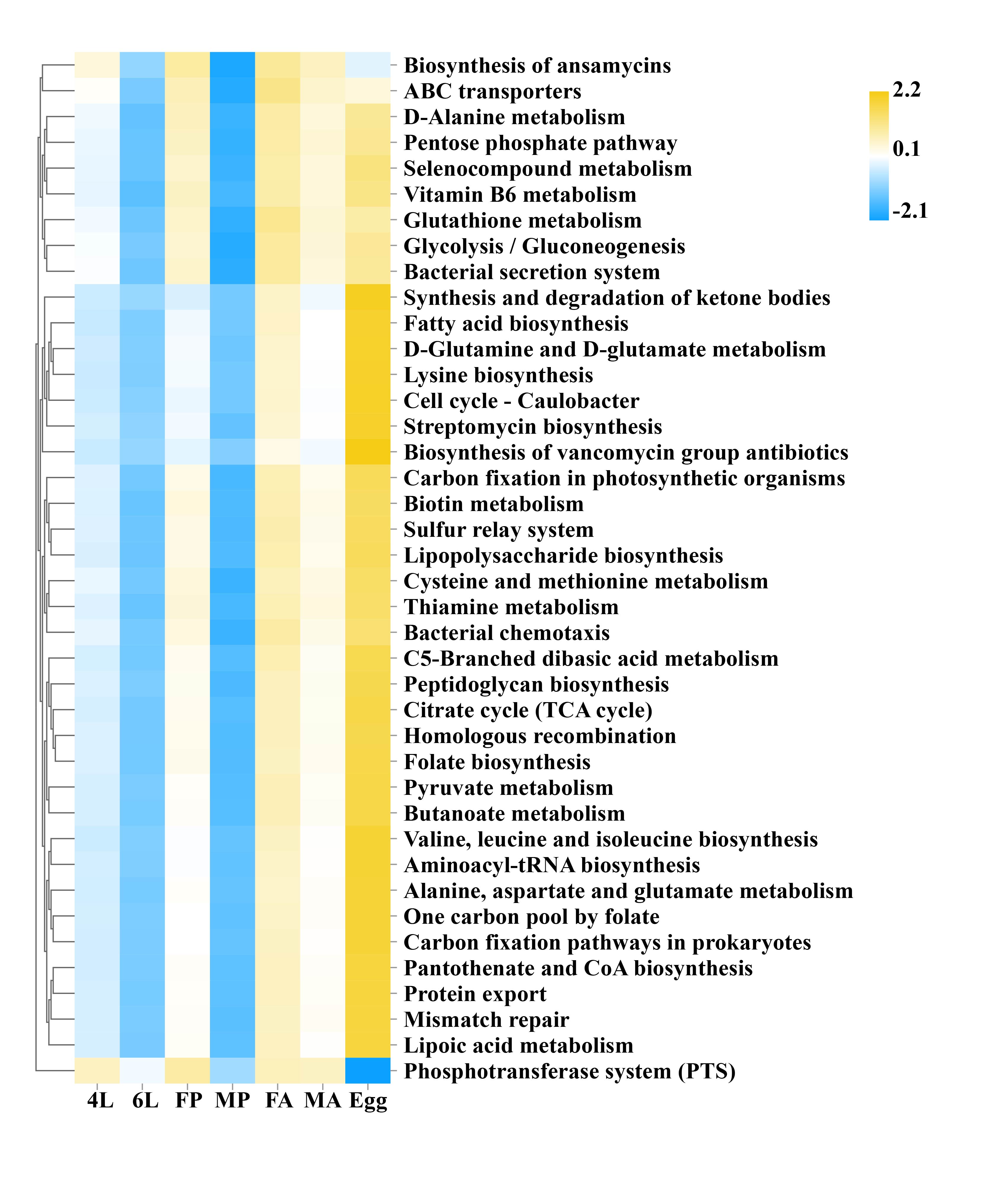

Supplement: Supplementary file 3 [file Image_3.JPEG]
