# Supplementary material for: Composition and diversity of gut microbiota across developmental stages of Spodoptera frugiperda and its effect on the reproduction
Source: Front Microbiol. 2023 Sep 18;14:1237684. doi: 10.3389/fmicb.2023.1237684 (PMC10543693; doi:10.3389/fmicb.2023.1237684)
Supplement: Supplementary file 4 [file Table_1.DOCX]

**SUPPLEMENTARY MATERIAL**

**Table S1 16S rRNA sequencing data**

**Figure S1 Rarefraction curves based on alpha-diversity.** (A) Chao1;(B) Shannon;(C) Good’s coverage; (D) Ananlysis of Good’s coverage by t test (p<0.05). 4L: 4th instar; 6L: 6th instar; MP, male pupa; FA, female adult; MA, male adult; FP, female pupa.

**Figure S2 Differences in the average OTU number at different life stages.** 4L: 4th instar; 6L: 6th instar; MP, male pupa; FA, female adult; MA, male adult; FP, female pupa.

**Table S2 The total amount of sequences generated from each stage.**

**Table S3 Tags of the top 12 common genera and the corresponding OTUs at different developmental stages.** 4L: 4th instar; 6L: 6th instar; MP, male pupa; FA, female adult; MA, male adult; FP, female pupa.

**Figure S3 Inferred functions** **of bacterial communities associated with *Spodoptera frugiperda*.** The heatmap of Kyoto Encyclopedia of Genes and Genomes (KEGG) level-3 functions of bacteria during various developmental stages of *Spodoptera frugiperda*. 4L: 4th instar; 6L: 6th instar; MP, male pupa; FA, female adult; MA, male adult; FP, female pupa.

**Figure S4** **Comparative analysis of phenotypic differences of bacterial communities associated with *Spodoptera frugiperda*.** 4L: 4th instar; 6L: 6th instar; MP, male pupa; FA, female adult; MA, male adult; FP, female pupa. *, P < 0.05; **, P < 0.0; ***, P < 0.001; ****, P < 0.0001.

**Table S1**

| **Category** | **Number** |
| --- | --- |
| Total of Raw Reads | 2761310 |
| Total of Clean Reads | 2758386 |
| OTUs | 3292 |
| Phyla | 25 |
| Classes | 43 |
| Orders | 81 |
| Families | 130 |
| Genera | 259 |

**Table S2**

| **ID** | **Genus** | **4L** | **6L** | **FP** | **MP** | **FA** | **MA** | **Egg** |
| --- | --- | --- | --- | --- | --- | --- | --- | --- |
| OTU000004 | *Sediminibacterium* | 1563 | 1265 | 1491 | 1429 | 5329 | 3945 | 27752 |
| OTU000002 | *Ralstonia* | 1383 | 1718 | 1639 | 1492 | 11427 | 3908 | 25409 |
| OTU000005 | *Acinetobacter* | 1403 | 230 | 62 | 67 | 1105 | 993 | 5999 |
| OTU000008 | *Bradyrhizobium* | 354 | 349 | 259 | 334 | 1648 | 1264 | 5111 |
| OTU000009 | *Brevundimonas* | 187 | 114 | 140 | 235 | 947 | 446 | 2878 |
| OTU000010 | *Pseudomonas* | 366 | 43 | 47 | 23 | 77 | 95 | 2493 |
| OTU000012 | *Prauserella* | 152 | 103 | 90 | 78 | 481 | 372 | 2186 |
| OTU000011 | *Rubrobacter* | 118 | 118 | 69 | 98 | 217 | 313 | 2034 |
| OTU000001 | *Enterococcus* | 70608 | 91854 | 53940 | 89152 | 53002 | 61290 | 1118 |
| OTU000016 | *Alteribacillus* | 59 | 52 | 39 | 20 | 184 | 178 | 948 |
| OTU000003 | *Enterobacter* | 36943 | 18913 | 43440 | 1804 | 39223 | 36166 | 696 |
| OTU000007 | *Providencia* | 307 | 654 | 13881 | 25567 | 6828 | 10870 | 243 |
